# GPR50 Interacts with TIP60 to Modulate Glucocorticoid Receptor Signalling

**DOI:** 10.1371/journal.pone.0023725

**Published:** 2011-08-17

**Authors:** Jian Li, Laura E. Hand, Qing-Jun Meng, Andrew S. I. Loudon, David A. Bechtold

**Affiliations:** Faculty of Life Sciences, University of Manchester, Manchester, United Kingdom; Ecole Normale Supérieure de Lyon, France

## Abstract

GPR50 is an orphan G-protein coupled receptor most closely related to the melatonin receptors. The physiological function of GPR50 remains unclear, although our previous studies implicate the receptor in energy homeostasis. Here, we reveal a role for GPR50 as a signalling partner and modulator of the transcriptional co-activator TIP60. This interaction was identified in a yeast-two-hybrid screen, and confirmed by co-immunoprecipitation and co-localisation of TIP60 and GPR50 in HEK293 cells. Co-expression with TIP60 increased perinuclear localisation of full length GPR50, and resulted in nuclear translocation of the cytoplasmic tail of the receptor, suggesting a functional interaction of the two proteins. We further demonstrate that GPR50 can enhance TIP60-coactiavtion of glucocorticoid receptor (GR) signalling. In line with *in vitro* results, repression of pituitary *Pomc* expression, and induction of gluconeogenic genes in liver in response to the GR agonist, dexamethasone was attenuated in *Gpr50^−/−^* mice. These results identify a novel role for GPR50 in glucocorticoid receptor signalling through interaction with TIP60.

## Introduction

GPR50 (G protein coupled receptor 50) shares approximately 45% identity in amino acid sequence with the melatonin receptors MT1 and MT2 [1], and has been identified as a mammalian orthologue of the avian/amphibian Mel1c receptor [2]; yet it does not bind melatonin [3] and remains an orphan receptor. Our previous studies implicate GPR50 in hypothalamic control of energy expenditure and feeding behaviour [4], [5]. Specifically, *Gpr50* expression in the brain is highly responsive to energy status being decreased by both fasting and high fat diet feeding [5], and *Gpr50^−/−^* mice demonstrate elevated metabolic rate, reduced fat accumulation, and partial resistance to diet-induced obesity. In humans, polymorphisms in *Gpr50* have been linked to elevated circulating triglycerides and cholesterol levels [6], as well as psychiatric affective disorders including bipolar disorder [7], [8].

Little is known about the intracellular signalling pathways downstream of GPR50. It has been suggested that GPR50 may participate in G-protein independent signalling, possibly involving cleavage of its intracellular carboxy-terminal domain [1], [9]. The c-terminal tail domain of GPR50 is one of the longest among mammalian GPCRs, and contains at least one putative proteolytic cleavage site [1]. GPR50 was recently shown to heterodimerise with the MT1 receptor and block melatonin-dependent signalling [9]. Interestingly, the intracellular tail of GPR50 is essential for this inhibition.

In the present study, we employed a yeast two-hybrid system to screen for potential binding partners of GPR50. Using the intracellular c-terminal domain of GPR50 as bait, this screen revealed an association of the receptor with the HIV-1 tat interactive protein, TIP60 (gene name *Kat5*). TIP60 is a transcriptional co-activator with histone acetyltransferase (HAT) activity, which has been implicated in the regulation of transcription, DNA repair and apoptosis [10], [11]. Among other interactions, TIP60 enhances the transcriptional activity of a range of transcription factors including nuclear hormone receptors (NHR)[12]. Here we confirm the association of GPR50 and TIP60 using immunoprecipitation and co-localisation, and go on to demonstrate a functional significance of this interaction *in vitro* and *in vivo*.

## Results

### Identification of TIP60 as an interaction partner of GPR50

**Figure 1:**
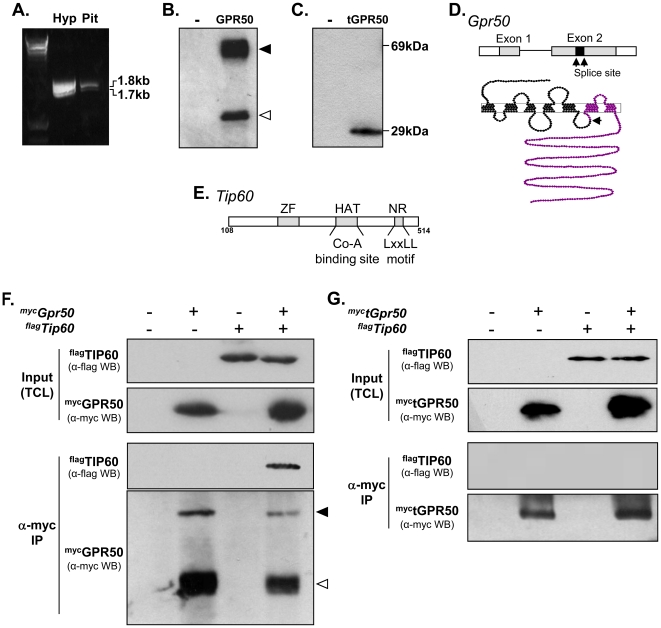
Association of GPR50 with TIP60. (**A–D**) Identification and cloning of full length and truncated GPR50. RT-PCR cloning of *Gpr50* from mouse hypothalamus (hyp) and pituitary (pit) produced two bands, reflecting full length *Gpr50* (∼1.8 kb) and a novel truncated transcript (∼1.7 kb; tGPR50; cDNA ladder shown in the first lane) (**A**). Western blotting of ^myc^GPR50 (**B**) and ^myc^tGPR50 (**C**) in HEK293 cells using an anti-myc antibody revealed proteins of the expected size (∼69 kDa for full length, ∼29 kDa for the truncated form). Filled and open arrowheads indicate the full-length and putative cleavage fragment of GPR50, respectively (**B**). Schematic of *Gpr50* gene (black arrows indicate splicing sites) and GPR50 protein (purple indicates the portion of the protein lost in the truncated version of the receptor) (**D**). (**E–G**) Identification and verification of TIP60 as a binding partner of GPR50. Schematic representation of the region of TIP60 encoded by the positive interacting clones identified in yeast two hybrid screen. This region includes the zinc finger (ZF), histoneacetyltransferase (HAT) and nuclear hormone receptor (NHR) binding domains [32](**E**). HEK293 cells were transfected with *^flag^Tip60*, or *^myc^Gpr50*, or both (as indicated), and total cell lysates (TCL) were subject to immunoprecipitation (*IP*) using anti-myc antibody. Western blotting (*WB*) of immunoprecipitates using anti-Flag antibody, confirmed the specific association of ^flag^TIP60 with ^myc^GPR50 (**F**). Full-length and the c-terminal cleavage fragment of GPR50 were detected in the immunoprecipitate (filled and open arrowheads, respectively). No precipitation of TIP60 was observed when truncated GPR50 was used for IP (**G**).

Cloning of full length *Gpr50* from mouse hypothalamus and pituitary by RT-PCR identified two transcripts in each tissue (Figure 1A). DNA sequencing confirmed the larger transcript to be full length *Grp50*, while the lower band consisted of a splice variant of *Gpr50*, in which a fragment of exon 2 had been removed (139 base pair deletion between nucleotides 720–860). The altered splicing introduces a premature stop-codon, which results in a truncated form of GPR50 protein (herein referred to as tGPR50) lacking the last two transmembrane regions and cytoplasmic domain (Figure 1A–D). Western blot analysis of c-terminal myc-tagged full-length GPR50 (Figure 1B) and tGPR50 (Figure 1C) confirmed the expression of both forms (∼69 kDa and ∼29 kDa, respectively). Interestingly, immunoblotting of full-length GPR50 also revealed a lower molecular weight band (∼35 kDa; Figure 1B open arrowhead), which likely reflects a proteolitic cleavage product of the c-terminal domain.

To identify novel binding partners for GPR50, we used a yeast two-hybrid system to screen mouse testes cDNA library, with the c-terminal domain of the receptor (cGPR50) as bait. Eleven potential interaction partners of GPR50 were identified (Table 1), and among these we focused on the transcriptional co-activator, TIP60. The interaction of GPR50 occurred within a region of TIP60 that included its zinc finger motif, HAT region, and LXXLL motif (Figure 1E). By using sequential transformation in yeast cells, we confirmed the interaction of full length TIP60 with the cytoplasmic domain of GPR50, while no interaction with the truncated form of GPR50 was observed.

**Table 1:** Results of Yeast Two-Hybrid Screen.

Gene name	Growth on selection plate(Trp^-^Leu^-^His^-^Ade^-^)	β-galactosidase Activity		Number of Positive clones
		white	blue	
Tip60	_+_		_+_	2
Cortexin 1	_+_		_+_	1
Sorting nexin 5	_+_		_+_	3
Sorting nexin 6	_+_		_+_	1
Kinesin 9	_+_	_+_		1
A kinase anchor protein 10	_+_	_+_		1
Syndecan binding protein 8	_+_		_+_	1
Gametogentin	_+_		_+_	5
Spermatogenesis	_+_	_+_		1
TBC1domain member 19	_+_		_+_	1
Peroxin 2	_+_		_+_	1

Following the yeast two-hybrid screen, the association between GPR50 and TIP60 was further confirmed in mammalian cells by co-immunoprecipitation. Here, HEK293 cells were transfected with full-length myc-tagged GPR50, full-length flag-tagged TIP60, or both constructs. Immunoprecipitation with an anti-myc antibody resulted in ^flag^TIP60 precipitation in cells co-transfected with ^myc^GPR50 (Figure 1F), but not in cells expressing ^flag^TIP60 alone or in combination with truncated GPR50 (Figure 1G), indicating the specific association of these two proteins.

### Association with TIP60 drives cytoplasmic and nuclear localisation of GPR50

**Figure 2:**
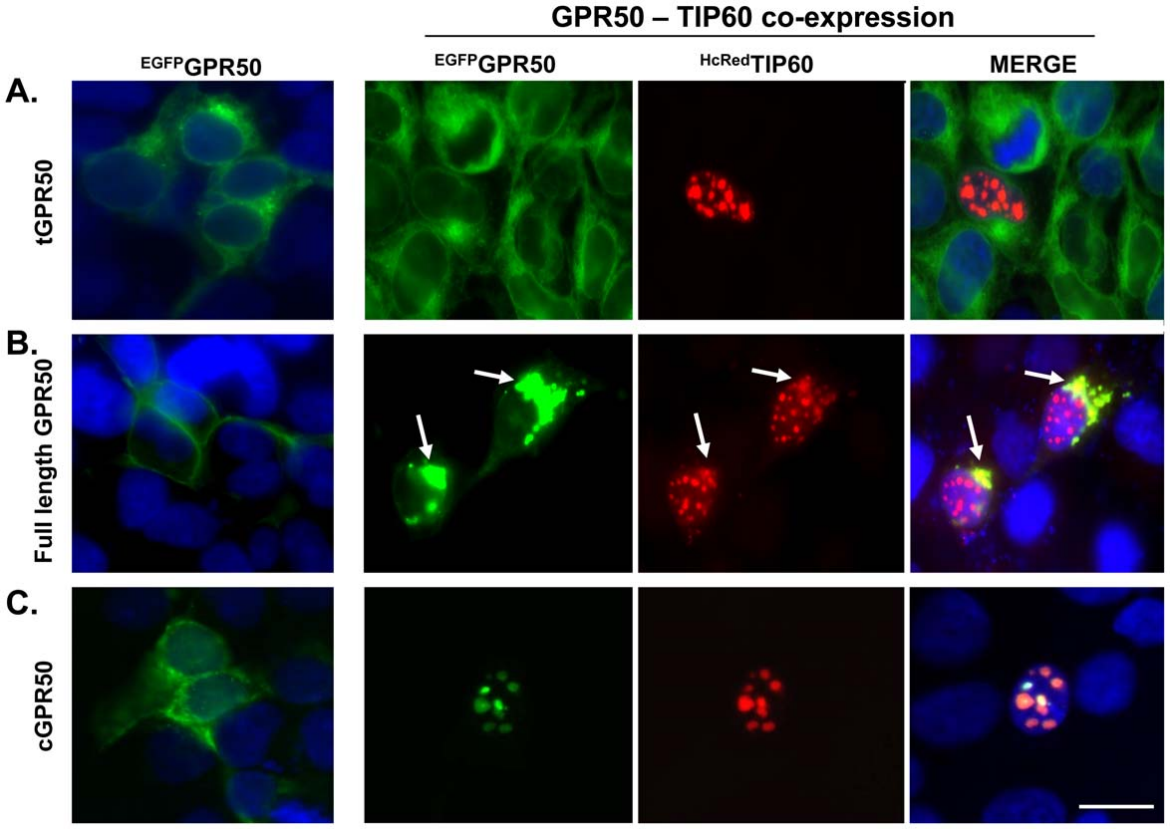
Subcellular localization of GPR50 and TIP60 in mammalian cells. ^EGFP^GPR50, ^EGFP^tGPR50, and ^EGFP^cGPR50 were expressed alone or in combination with ^HcRED^TIP60 in HEK293 cells. When expressed alone (far left panels), full length GPR50 localised predominantly to the cell membrane (**B**), while tGP50 (**A**) and cGPR50 (**C**) remained cytoplasmic. ^HcRED^TIP60 was confined to the nucleus when expressed with tGPR50 (**A**). In contrast, co-expression of TIP60 with full length GPR50 resulted in a pronounced perinuclear localisation of both proteins (**B**, arrows), while co-expression with cGPR50 led to nuclear compartmentalisation of the receptor tail (**C**). Blue  =  DAPI counterstaining; Magnification Bar  = 20 µm.

Using fluorescently-tagged proteins, we examined the subcellular localisation patterns of GPR50, tGPR50, and cGPR50 in the presence or absence of TIP60 in HEK293 cells. When expressed alone, full length GPR50 localised primarily to the cell membrane, while the truncated form and c-terminal tail remained in the cytoplasm (Figure 2, far left panels). As expected, TIP60 was localised exclusively within the nucleus when expressed alone (not shown) or when co-expressed with tGPR50 (Figure 2A). In contrast, co-expression of TIP60 with full length GPR50 resulted in a pronounced perinuclear localisation of both proteins, although GPR50 localisation to the plasma membrane was still observed (Figure 2B). Interestingly, when co-expressed with TIP60, the c-terminal tail (cGPR50) was translocated to the nuclear compartment where it co-localised with TIP60 (Figure 2C). These findings demonstrate that the association of GPR50 and TIP60 alters the cellular compartmentalisation of both proteins, and likely represents a functional interaction.

### GPR50 modulates TIP60-dependent GR signalling

**Figure 3:**
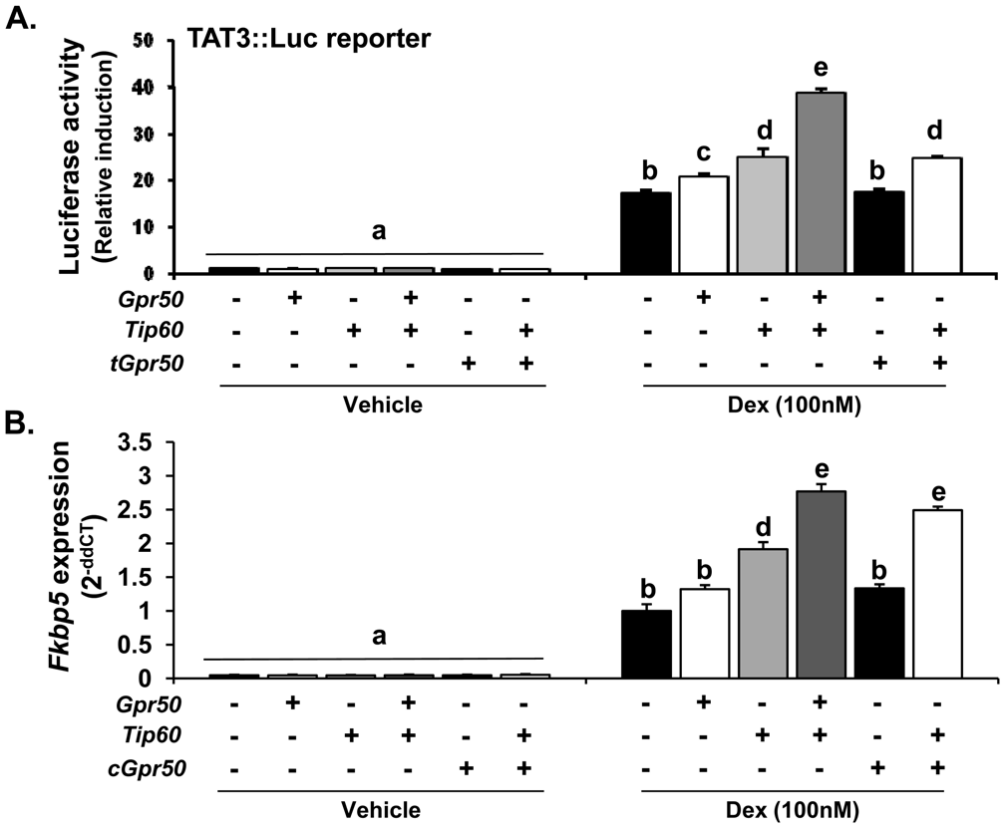
Functional interaction of TIP60 and GPR50 on GR signalling. (**A–B**) The impact of GPR50 on TIP60-mediated GR signalling was assayed in HEK293 cells using a luciferase-based transcriptional reporter for GR (TAT3::luc)(**A**) and quantitative RT-PCR of a GR-responsive target gene (Fkbp5)(**B**). Full-length *Gpr50*, *tGpr50, cGpr50* and/or *Tip60* constructs were transfected alone or in combination as indicated.

A role for TIP60 in enhancing transcriptional activity of NHRs is well-established [12]. We therefore examined the functional impact of the GPR50 and TIP60 interaction on GR-mediated gene expression, using a luciferase-based transcriptional reporter assay for GR activity (TAT3::luc [13], and the endogenous expression of the known GR-target gene, FK506-binding protein 5 (Fkbp5; [14]) (Figure 3). Dexamethasone (Dex, 100 nM) caused a significant increase in TAT3::luc activity in HEK293 cells, which was further enhanced by the expression of either GPR50 or TIP60 (Figure 3A). Importantly, co-expression of both GPR50 and TIP60 resulted in a significant potentiation of Dex-induced reporter activity when compared to either single expression, suggesting a synergistic effect of the two proteins in modulating GR signalling. The truncated form of GPR50 did not alter luciferase activity when expressed alone or in combination with TIP60 (Figure 3A). The ability of TIP60 and GPR50 to enhance GR-mediated expression of an endogenous gene was confirmed by QPCR analysis of Fkbp5 expression (Figure 3B). Over-expression of TIP60 increased Fkbp5 transcription, with a further significant potentiation observed upon co-expression of TIP60 with either full-length GPR50 or the cytoplasmic tail of GPR50.

**Figure 4:**
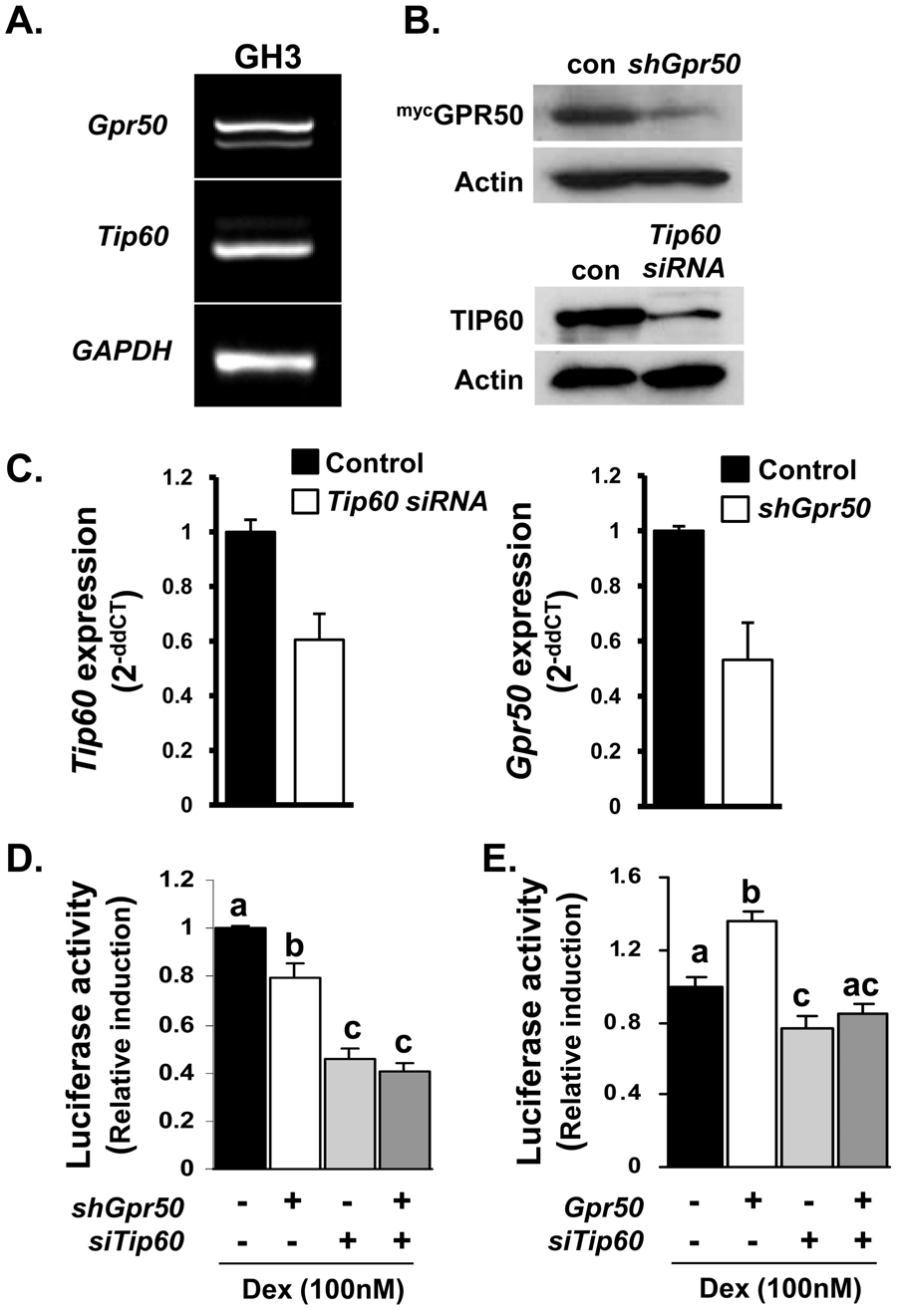
Endogenous TIP60 and GPR50 modulate GR signalling in GH3 cells. (**A–D**) Contribution of endogenous GPR50 and TIP60 to GR-signalling in GH3 cells. Expression of both *Gpr50* and *Tip60* was confirmed in GH3 cells (**A**). *shGpr50* or *siTip60* were efficient at attenuating the expression of their respective targets as demonstrated by reduced protein accumulation following transient transfection (**B**) and reduced levels of endogenous mRNA transcript (**C**). Importantly, knockdown of either GPR50 or TIP60 in GH3 cells attenuated the induction of TAT3::luc activity in response to Dex (100 nM), although no additive effect was observed with combined knockdown of both proteins (**C**). The potentiation of Dex-induced TAT3::luc activity by *Gpr50* over-expression was also blocked by knockdown of endogenous TIP60 using *siTip60* (**D**). Differences in lettering reflect statistically significant differences between treatment groups (two-way ANOVA, with Bonferroni's post hoc test). Data are representative of 3 independent experiments, each performed in triplicate.

As GPR50 remains an orphan receptor, we cannot manipulate its activity directly to assess the contribution of endogenously expressed receptor on GR or TIP60 function. We have therefore employed a genetic knockdown strategy in a pituitary cell line (GH3). We first confirmed that both *Gpr50* and *Tip60* are endogenously expressed in the GH3 cells (Figure 4A), and that transfection with *shGpr50* or *siTip60* was effective in attenuating the expression of their respective genes. Knockdown was shown to decrease protein expression in cells over-expressing tagged GPR50 and TIP60 (Figure 4B), as well as attenuate endogenous mRNA expression (Figure 4C). Importantly, knockdown of either endogenous GPR50 or TIP60 attenuated Dex-induced TAT3::luc luciferase activity in GH3 cells (Figure 4D). Combined knockdown of GPR50 and TIP60 did not, however, decrease luciferase activity below that achieved with *siTip60* alone. Moreover, knockdown of endogenous *Tip60* expression abolished the ability of *Gpr50* over-expression to enhance Dex-induced luciferase activity in the GH3 cells (Figure 4D). Together these studies demonstrate that endogenously expressed *Gpr50* and *Tip60* contribute to GR-mediated signalling in pituitary cells, and that TIP60 is required for GPR50 modulation of GR-signalling.

### Altered GR responses in *Gpr50^−^* ^/−^ mice

**Figure 5:**
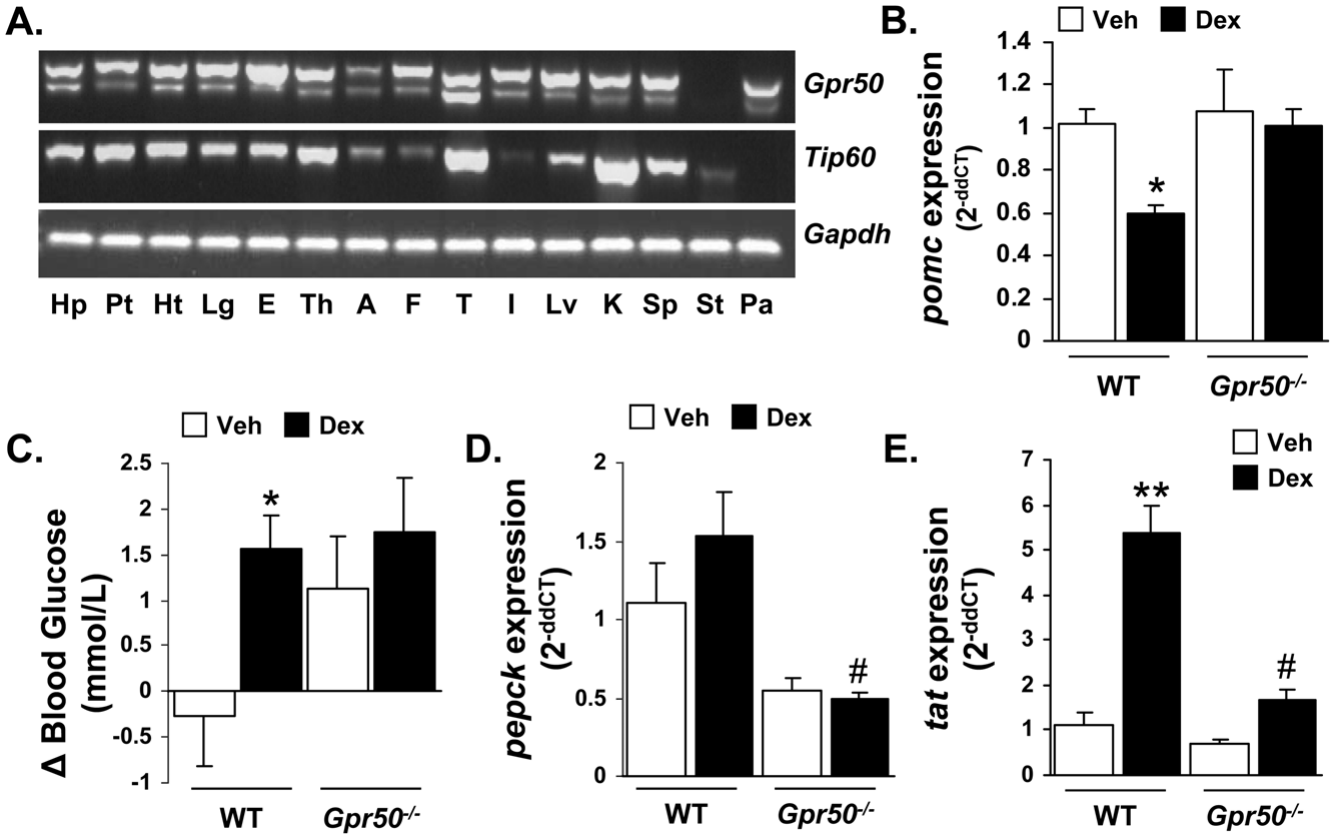
Altered dexamethasone responses in *Gpr50^−/−^* mice. (**A**) RT-PCR profiling of *Gpr50* and *Tip60* mRNA expression in mouse tissues. Hp, hypothalamus; Pt, pituitary; Ht, heart; Lg, lung; E, eye; Th, thyroid; A, adrenal; F, white fat; T, testes; I, intestine; Lv, liver; K, kidney; Sp, spleen; St, stomach; Pa, pancreas. (**B–E**) The effects of dexamethasone (Dex, 0.1 mg/kg) were examined in WT and *Gpr50^−/−^* mice, in terms of glucocorticoid feedback and glucose homeostasis. *Pomc* mRNA expression in the pituitary was reduced 5 hr after Dex treatment in WT, but not *Gpr50^−/−^* mice (**B**). Circulating blood glucose was significantly increased in response to Dex only in WT mice (**C**). Similarly, *Gpr50^−/−^* mice exhibited an attenuated induction of the liver gluconeogenic genes *Pepck* (**D**) and *Tat* (**E**) in response to Dex. Gene expression has been normalised to vehicle treated levels, and blood glucose presented as change from time 0 to 5 h post-injection. *  =  P<0.05, **  =  P<0.01 versus vehicle treatment within genotype, #  =  P<0.05 versus WT Dex treatment, two-way ANOVA with Bonferroni's post hoc test. Data representative of two independent experiments *n* = 5 mice/group in each experiment for **B–D**.

*Gpr50* and *Tip60* exhibit widespread and overlapping expression profiles in mouse tissues (Figure 5A), indicating the potential for functional interaction of the proteins *in vivo*. We have previously reported that mice lacking *Gpr50* exhibit elevated circulating corticosterone [5], which combined with our current *in vitro* demonstration of GPR50 potentiation of GR signalling, suggests that glucocorticoid feedback may be diminished in these mice. We therefore tested the effect of Dex administration on the expression of gluconeogenic genes in the livers and proopiomelanocortin (*pomc*; the source of ACTH) in the pituitaries of wild-type (WT) and *Gpr50^−/−^* mice. Dex (0.1 mg/kg ip) significantly reduced pituitary expression of *pomc* in WT, but not *Gpr50^−/−^* mice 5 hr post-administration (Figure 5B). Despite this difference, circulating corticosterone levels were reduced significantly by Dex in both genotypes, demonstrating that pituitary *pomc* expression in the pituitary is not the only level of negative glucocorticoid feedback onto the hypothalamic-pituitary-adrenal (HPA) axis. Stimulation of the gluconeogenic pathways by Dex was also blunted in *Gpr50^−/−^* mice. Specifically, circulating blood glucose and the expressions of phosphoenolpyruvate carboxykinase (*Pepck*) and tyrosine aminotransferase (*Tat*) in the liver were elevated by dex-administration in WT mice, whereas no significant increase was observed for any of the three measures in *Gpr50^−/−^* mice (Figure 5C–E). Taken together these results suggest that GR signalling is diminished in mice lacking GPR50, and support *in vitro* evidence that GPR50 can modulate GR function.

## Discussion

GPR50 is a member of the rhodopsin-like subclass of GPCRs, and is the mammalian orthologue of the avian/amphibian melatonin receptor, Mel1c [2]. Yet during its evolution GPR50 lost the ability to bind melatonin, and to date no ligand has been identified, and the G-proteins with which GPR50 couples have not been defined. During its divergence from the melatonin receptors, GPR50 acquired a long intracellular tail domain. It has been proposed that this tail mediates GPR50 function; for example by blocking the signalling of other GPCRs during receptor heterodimerisation [9], or through proteolytic cleavage to produce soluble signalling proteins [1], [9]. For this reason, we sought to define potential intracellular interaction partners of GPR50. As a result, we have identified and confirmed a functional association between GPR50 and the transcriptional co-activator, TIP60.

TIP60 has been shown to modulate the transcriptional activity of a variety of transcription factors, including NHRs, and can act as both transcriptional enhancer and repressor dependent on its target transcription factor [15]. TIP60 facilitates the recruitment of protein complexes to the transcriptional machinery, and possesses intrinsic HAT activity, having been shown to acetylate both histones (H2A, H3, H4)[11], [16] and non-histone proteins such as the androgen receptor (AR)[17]. TIP60 exhibits wide cellular and tissue distribution, and has been implicated in a number of physiological processes, including DNA repair, apoptosis, adipogenesis, as well as NF-κB and p53 signalling, likely due to the diversity of transcription factors with which it associates [15], [18]. Therefore, the ability of GPR50 to interact with TIP60 implicates this receptor in numerous physiological processes.

The physical interaction of GPR50 with TIP60 may initially seem counter-intuitive due to differences in the sub-cellular compartmentalisation of the two proteins (i.e. membrane bound versus nuclear). However, as we demonstrate with cellular localisation studies, co-expression of GPR50 and TIP60 alters the localisation of both proteins. Specifically, full-length receptor associated with TIP60 within the perinuclear space, while the cytoplasmic tail of GPR50 was translocated into the nucleus. This type of interaction is not without precedent. Cytoplasmic and perinuclear localisation of TIP60 has been reported previously [19], and it has been shown to interact with other membrane bound receptors [20], [21]. For example, internalisation of the endothelin receptor (also a G-protein coupled receptor) in response to stimulation leads to an increase of TIP60 in the perinuclear region and functional interaction of the two proteins [20], similar to that observed here. Increased perinuclear TIP60 content may not require nuclear efflux, as TIP60 is a relatively unstable protein with a short half-life (∼30–190 min)[22]; it is possible that newly synthesised protein could associate with the receptors prior to nuclear translocation. As the ligand of GPR50 is unknown, we cannot assess the degree to which ligand binding might impact on GPR50 internalisation or association with TIP60. Importantly, our genetic knockdown studies demonstrate that endogenous GPR50 and TIP60 interact to modulate GR-signalling. One intriguing possibility is that the intracellular tail of GPR50 is specifically cleaved in response to an extracellular (or intracellular) signal, liberating it to associate with TIP60 and translocate to the nucleus. In support of this, Western blot analysis of cells expressing full-length GPR50 containing a c-terminal myc-tag, revealed a c-terminal cleavage product (∼35 kD).

As mentioned, a role for TIP60 in enhancing the transcriptional activity of NHRs is well-established [12], and here we have assessed the impact of GPR50 on GR signalling. Interestingly, the influence of GPR50 on TIP60-mediated NHR transcriptional activity may differ depending on the specific NHR target. Our preliminary studies suggest that GPR50 attenuates TIP60-mediated enhancement of PPARγ signalling (Figure S1). Divergent effects of GPR50 on TIP60/NHR interactions may be due to the physical interaction of the three proteins. TIP60 typically associates with the ligand-binding domain of NHRs through a conserved NHR-binding motif (LXXLL) located in its c-terminal domain. These residues are critical for the association of TIP60 with AR, estrogen receptor (ER) and GR, yet not for its binding of PPARγ [12], [23], [24]. Further, unlike GR, the association of TIP60 with PPARγ can occur in the absence of ligand binding. Ligand-independent signalling of PPARγ involving TIP60 was also observed in the current study (Figure S1). It is therefore possible that binding of GPR50 masks the site at which TIP60 binds PPARγ, or blocks the binding of other associated proteins required for TIP60/PPAR signalling.

Importantly, in support of the *in vitro* demonstration that GPR50 enhances GR signalling, we demonstrate that responses to Dex are attenuated *in vivo,* in *Gpr50^−/−^* mice in terms of glucocorticoid regulation of *pomc* expression in pituitary and gluconeogenic genes in the liver. The HPA axis regulates corticosterone release from the adrenal gland, and is subject to negative regulatory feedback at multiple levels, such as repression of pituitary *pomc* expression by corticosterone. This response was diminished in *Gpr50^−/−^* mice in response to dex administration, which is in line with the prevalent expression of both GPR50 and TIP60 in the pituitary. The significant decrease in circulating corticosterone in the *Gpr50^−/−^* mice despite the attenuated *pomc* response likely reflects continued negative feedback at another level of the HPA, such as at the adrenal itself. mRNA expression profiling demonstrates that TIP60 and GPR50 are both expressed in a wide array of tissues suggesting that there is ample opportunity for the two proteins to interact *in vivo*, although it will now be important to examine cellular expression in detail to confirm co-expression within these tissues.

We have previously reported that *Gpr50^−/−^* mice exhibit reduced weight gain, elevated metabolic rate and partial resistance to diet-induced obesity [5]. Further, *Gpr50* expression in the brain is highly responsive to energy status being decreased by both fasting and high fat diet feeding [5]. This implies that the influence of GPR50 on TIP60-mediated signalling may itself be responsive to the energy status of the organism. TIP60 has been implicated in glucose homeostasis [25] and adipogenesis [24]. Further, TIP60 can repress the activity of the transcription factor signal transducer and activator of transcription 3 (STAT3) [26], a major downstream target of leptin signalling [27]. Alterations in leptin response may be an important aspect to the altered metabolism and feeding behaviour observed in the *Gpr50^−/−^* mice [5].

In summary, the current study reveals a novel role for GPR50 in modulating TIP60 transcriptional activity, and as a result GR signalling. The interaction of GPR50 with TIP60 was initially identified using a yeast two hybrid screen, and subsequently confirmed by immunoprecipitation and co-localisation within mammalian cells. Importantly, the functional impact of GPR50 on GR signalling is supported by genetic knockdown in pituitary cells and *in vivo* studies using *Gpr50^−/−^* mice. The possibility that proteolytic cleavage of the c-terminal tail of GPR50 produces functional signalling proteins is intriguing and merits further investigation.

## Materials and Methods

### Expression constructs

For the yeast two-hybrid screening and the following growth assay, the coding sequences of the cytoplasmic domain of mouse GPR50 (amino acids 304–592, cGPR50), truncated GPR50 (amino acids 1–264, tGPR50), and full-length GPR50 (amino acids 1–592, GPR50) were amplified by RT-PCR from mouse hypothalamus, and cloned in-frame into pGBKT7 vector (Clontech, Saint-Germain-en-Laye, France), resulting in the pGBKT7-cGPR50, pGBKT7-tGPR50, and pGBKT7-GPR50 constructs, respectively. For expression in mammalian cells and immunoprecipitation assays, GPR50 were sub-cloned into pcDNA3-MycHis vector, resulting in ^myc^GPR50 and ^myc^tGPR50. ^flag^TIP60 construct was a kind gift from Professor John Lough (Medical College of Wisconsin, Wisconsin, USA)[28]. For cellular localisation studies, mouse TIP60 was sub-cloned downstream of the N-terminal HcRed sequence of the pHcRed-C1 vector (Clontech), and mouse GPR50 constructs cloned upstream of the enhanced green fluorescent protein (EGFP) sequence using the pEGFP-N1 vector (Clontech). The correct sequences and reading frames of all constructs derived from PCR products were verified by DNA sequencing. shRNA against *Gpr50* was purchased from Sigma (CCGGGCCAGCTCTAATCATCTTCATCTCGAGATGAAGATGATTAGAGCTGGCTTTTT, TRCN0000025780). Mission Nontarget shRNA Control Vector was used as control (Sigma). Validated siRNA against *Tip60* was purchased from QIAGEN (SI02780897). Negative Control siRNA was used as control (QIAGEN). Western blotting was performed to confirm knockdown efficiency.

### Yeast two-hybrid screening and growth assay

The yeast two-hybrid screening was conducted as previously described [29]. Briefly, a yeast strain (AH109) based on the Gal4 system was used, and transformations carried out using a standard lithium acetate method. For library screening, a sequential transformation was performed with the bait plasmid (pGBKT7-cGPR50) followed by 50 µg of mouse testis cDNA library (cloned into the pACT2 vector). The co-transformants were streaked onto appropriate selective media plates, as well as high stringency synthetic medium lacking histidine and adenine and containing X-gal. Interacting clones were selected by their abilities to grow or turn blue on appropriate selective plates. Plasmid DNA from positive yeast clones was rescued into bacterial strain KC8 (Clontech) and sequenced using a poly(T) sequencing primer. The resultant sequences were checked for similarity to known transcripts in the nucleotide sequence data bases using the BLAST algorithm. Phenotypes of yeast co-transformants on selective media were tested using standard yeast growth assay.

### Cell culture

HEK293 [30] and GH3 [31] cells were maintained in Dulbecco's modified Eagle's medium (DMEM, Sigma, Poole, UK) supplemented with 10% Fetal bovine serum (Sigma), penicillin/streptomycin (Gibco, Paisley, UK), and L-glutamine (Gibco). Cells were cultured at 37°C in a humidified 5% CO2 environment. For immunofluorescence, HEK293 cells were seeded on coverslips, followed by transfection, and fixation in 4% paraformaldehyde (24 h post-transfection).

### Immunoprecipitation and Western Blotting

Transfected HEK293 cells were lysed at 4°C for 30 min. The total cell lysates were pre-cleared with 20 µl of protein G-Sepharose (Sigma) for 1 h before they were immunoprecipitated overnight at 4°C with 50 µl of protein G-Sepharose and the anti-myc polyclonal antibody (MBL). The immunoprecipitated sample and 20 µl of each total cell lysate were separated on 12% polyacrylamide gels and blotted onto nitrocellulose membranes (Bio-Rad) for immunoblotting with anti-myc polyclonal antibody (1∶800; MBL, Woburn, USA) or anti-flag polyclonal antibody (1∶500; Sigma). Immunodetection were performed with the ECL Western blotting detection kit (Amersham Biosciences, Buckinghamshire, UK).

### RT-PCR and Real-time quantitative PCR

Total RNA isolated from mouse tissues were reverse transcribed and subjected to cDNA synthesis. Primer pairs were based on mouse sequences (5′ to 3′): *Gpr50*, ATGGCCAGCAGGCCTCTGCC (F) and TCACTCGCAGTAGAGCCCGT (R); glyceraldehyde-3-phosphate dehydrogenase (*Gapdh*), TCAACGGATTTGGTCGTAT (F) and ATGAGTCCTTCCACGATAC (R). Q–PCR was performed as described previously using the Platinum SyBR Green kit (Invitrogen). Mouse housekeeping gene *18S rRNA* was used as an internal control. Primer pairs were *Pomc*, GTGCCAGGACCTCACCAC (F), CTTCCGGGGGTTTTCAGT (R); *Fkbp5*, AGCCAAGGGTGACTTTGAGA (F), TCTGCAGTCTTGCAGCCTTA (R); *Pepck*, ACCTCCTGGAAGAACAAGGA (F), CTCATGGCTGCTCCTACAAA (R); *Tat,* CATCTGGAGCCATGTACCTT (F), TCCAGCATCATCACCTCG (R). *18S rRNA*, TCCGACCATAAACGATGCCGACT (F), TCCTGGTGGTGCCCTTCCGTCAAT (R); The reactions were performed in the ABI PRISM 7300 Sequence Detection System (Applied Biosystems, Warrington, UK).

### Luciferase reporter assay

The luciferase assay was performed as described previously [29]. Briefly, HEK293 cells were transiently transfected with 500 ng TAT3::luc or Fabp4::luc reporter constructs, together with ^flag^TIP60 and/or ^myc^GPR50 as indicated. TAT3::luc and Fabp4::luc constructs were generously provided by Dr. Laura Matthews (University of Manchester) and Dr. Susanne Mandrup (University of Southern Denmark). The TAT3-Luc reporter plasmid contain three copies of the tyrosine aminotransferase (TAT) GREs upstream of a minimal *Drosophila* alcohol dehydrogenase promoter (*adh* −33 to +53) and the luciferase gene [13]. 50 ng cytomegalovirus-renilla luciferase was included in all transfections to correct for transfection efficiency. 24 h after transfection, cells were treated as specified for 16 h (dexamethasone 100 nM, rosiglitazone 1 µM; Sigma). Cells were then lysed and assayed for luciferase activity using a dual-luciferase reporter assay system (Promega, Chilworth, UK). Data has been normalized to the bioluminescence recorded from mock transfected cells. All experiments were performed in triplicate and repeated 3 times.

### *In vivo* dexamethasone treatment and quantitative PCR

Congenic wild type (WT) and *Gpr50^−/−^* mice of C57B6 background were originally obtained from Organon Laboratories Ltd (Cambridge, UK) and subsequently bred at the University of Manchester. All research using animals was licensed under the Animals Act of 1986 (Scientific Procedures; Licence 40/3267) and received ethical approval from the University of Manchester animal welfare committee. Adult female mice (8–10 weeks of age) were used for all experiments, housed at an ambient temperature of 20–22°C, and maintained in a 12∶12-h light:dark lighting schedule. Leading up to experiments, animals were housed singly, and acclimated to handling and blood glucose measurement. Just prior to dex (0.1 mg/kg, ip) administration blood glucose was measured by tail prick using the MediSense optimum blood glucose sensor (MediSense, Oxon, UK). Blood glucose was measured again 5 h post-injection, following which trunk blood was collected for corticosterone analysis, and pituitary and liver removed onto dry-ice for subsequent Q-PCR analysis. Corticosterone was measured using an EIA kit (Cambridge BioScience Ltd, Cambridge, UK) according to manufacturer's instruction. Data shown for *in vivo* studies are representative of two independent cohorts of mice, with each study using 5 mice per group.

## Supporting Information

Figure S1 **Functional interaction of TIP60 and GPR50 on PPARγ signalling.** The impact of GPR50 on TIP60-mediated nuclear hormone receptor signalling was assayed in HEK293 cells using luciferase-based transcriptional reporters for PPARγ (Fabp4::luc). Co-transfection with *PPAR*γ, *Gpr50*, and *Tip60* constructs were performed as indicated. Histograms illustrate fold induction of Fabp4::luc activity following rosiglitazone (Ros, 1µM) treatment. Differences in lettering reflect statistically significant differences between treatment groups (two-way ANOVA, with Bonferroni's post hoc test). Data are representative of 3 independent experiments, each performed in triplicate.
(TIF)
