# Supplementary figures and images for: GPR50 Interacts with TIP60 to Modulate Glucocorticoid Receptor Signalling
Source: PLoS One. 2011 Aug 17;6(8):e23725. doi: 10.1371/journal.pone.0023725 (PMC3157439; doi:10.1371/journal.pone.0023725)

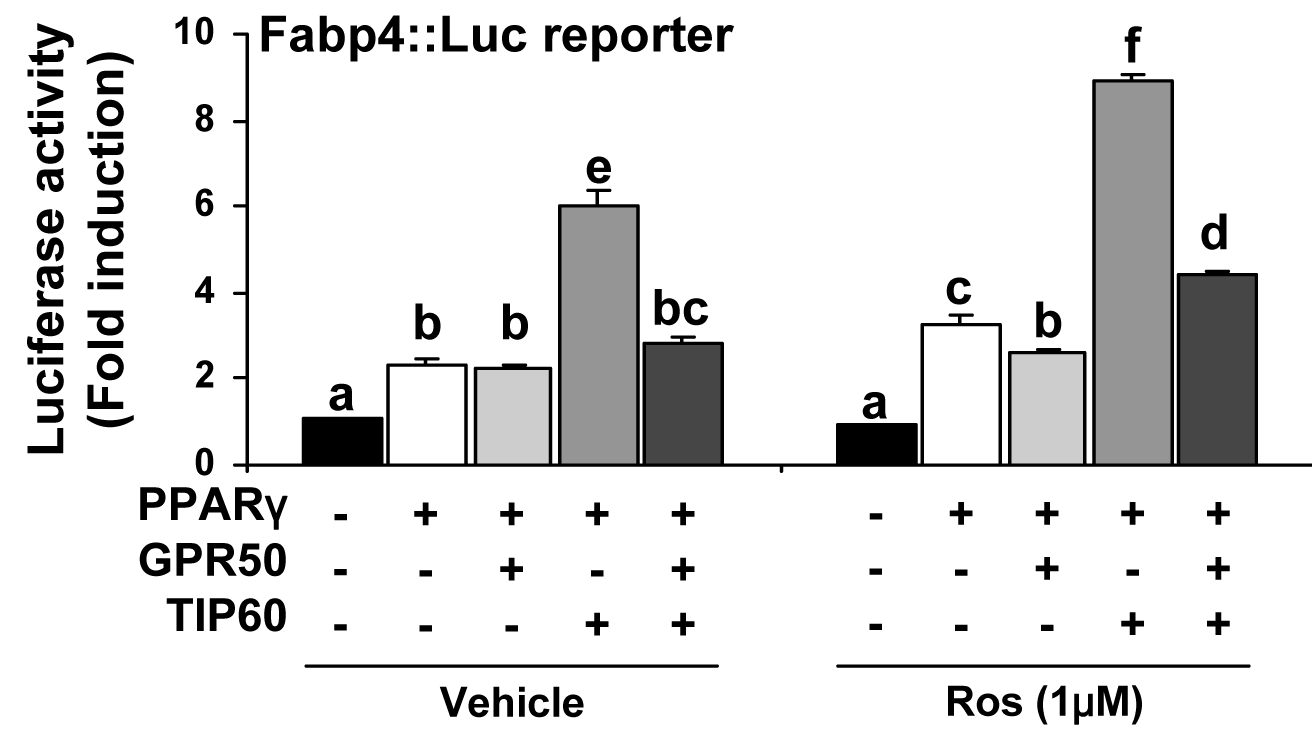

Supplement: Figure S1 — Functional interaction of TIP60 and GPR50 on PPARγ signalling. The impact of GPR50 on TIP60-mediated nuclear hormone receptor signalling was assayed in HEK293 cells using luciferase-based transcriptional reporters for PPARγ (Fabp4::luc). Co-transfection with PPARγ, Gpr50, and Tip60 constructs were performed as indicated. Histograms illustrate fold induction of Fabp4::luc activity following rosiglitazone (Ros, 1µM) treatment. Differences in lettering reflect statistically significant differences between treatment groups (two-way ANOVA, with Bonferroni's post hoc test). Data are representative of 3 independent experiments, each performed in triplicate. (TIF) [file pone.0023725.s001.tif]
